# Dr. Daniel Dwyer

**Published:** 1892-07

**Authors:** 


### Obituary.

### DR. DANIEL DWYER.

At the Twenty-eighth Annual Meeting of the Connecticut State Dental Association, the committee appointed to express the sentiments of the organization as regards the death of Dr. Daniel Dwyer reported as follows:

Whereas, This Society mourns the death of Daniel Dwyer, D.D.S., who died in Hartford, November 7, 1891.

Dr. Dwyer was one of the original members of this Society. He began the study of dentistry at an early age with Drs. Preston and McManus and with Dr. Crofoot, and was later for many years associated with Dr. J. M. Riggs. After years of active practice, he attended and graduated at the Baltimore Dental College.

Dr. Dwyer was a hard-working, conscientious dentist, and a warm-hearted, honest man.

*Resolved,* That the secretary be requested to place these resolutions on the records of the Society, and send a copy to his brother.

JAMES MCMANUS.
WILLIAM H. RIDER.
JOSEPH H. SMITH.

